# Deep venous thrombosis of the contralateral iliac vein after stenting of the iliocaval confluence: a therapeutic challenge

**DOI:** 10.1590/1677-5449.202201621

**Published:** 2023-06-30

**Authors:** Annata Teixeira Della Costa, Igor Rafael Sincos, Lorrane Vieira Siqueira Riscado

**Affiliations:** 1 Faculdade de Medicina de São José do Rio Preto, São José do Rio Preto, SP, Brasil.; 2 Universidade de São Paulo - USP, São Paulo, SP, Brasil.; 3 Universidade Federal de Juiz de Fora - UFJF, Juiz de Fora, MG, Brasil.

**Keywords:** deep venous thrombosis, angioplasty, May-Thurner syndrome, iliac vein

## Abstract

The treatment of choice for patients with symptomatic venous compression syndrome is venous stenting. However, this treatment has well-documented complications and, although rare, contralateral deep venous thrombosis is one of these complications. Our objective is to present a case of deep venous thrombosis of the contralateral iliac vein resulting from placement of the stent beyond the recommended position and the therapeutic challenge is to recanalize the vein with reconstruction of the iliocaval confluence.

## INTRODUCTION

May-Thurner Syndrome (MTS) is a relatively rare vascular condition in which the left common iliac vein (CIV) is compressed by the right common iliac artery. The condition may be asymptomatic, but when it is symptomatic, it provokes symptoms of chronic venous insufficiency (CVI) or proximal venous thrombosis, generally in young and middle-aged women. Proximal compression of the CIV is observed in combination with CVI in up to 55% of symptomatic patients.^1^

Venous stenting is the treatment of choice for patients with symptomatic CIV compression syndrome with CEAP classifications C3-C6.^2,3^ However, the stent must be implanted precisely, because if it is placed beyond the ideal position, into the inferior vena cava (IVC), it can compromise the contralateral venous flow and increase the risk of deep venous thrombosis (DVT).^4,5^

The objective of this report is to describe recanalization of the right CIV by pharmacomechanical thrombectomy and placement of a venous stent in the right common and external iliac veins in a patient with extensive lower limb (LL) DVT because of a prior stent positioned beyond the recommended 2 to 3 cm in the left CIV.

The protocol was approved by the institutional Research Ethics Committee (CAAE 60251422.8.0000.5226, consolidated opinion 5.506.965).

## PART I - CLINICAL CASE

**Figure 1:**
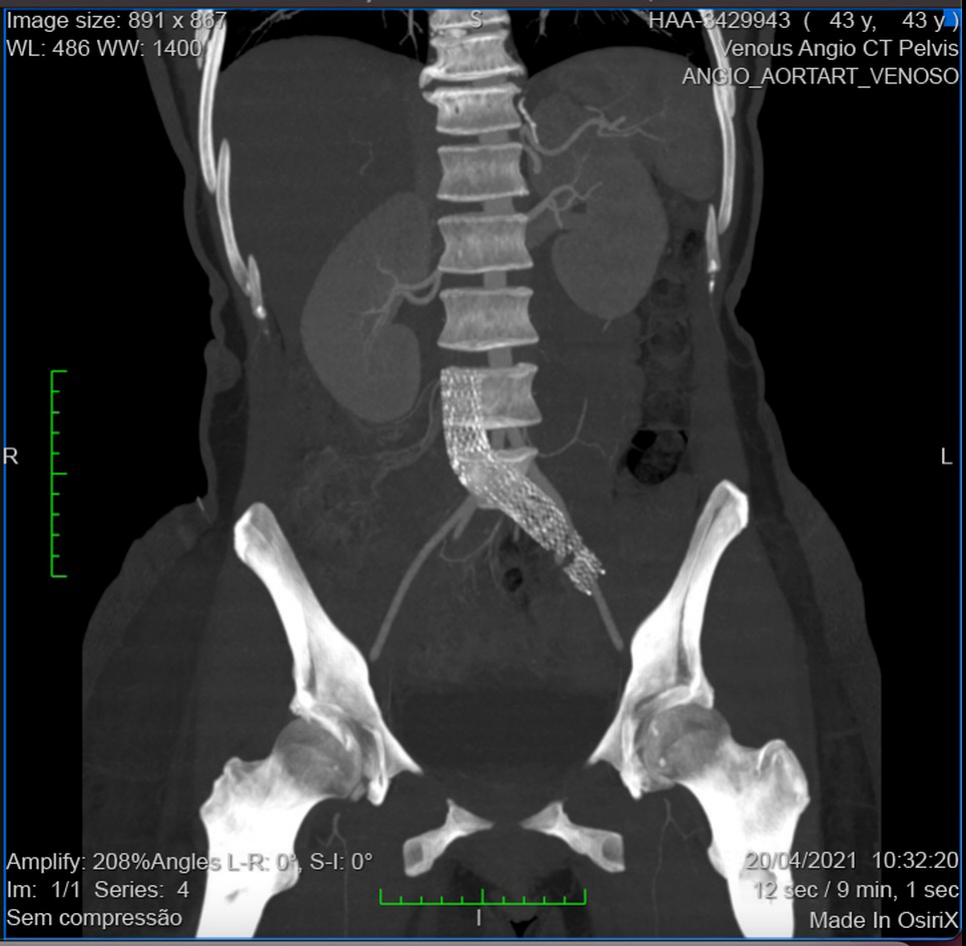
Computed tomography angiography showing the venous stent in the inferior vena cava and left common iliac vein.

A 43-year-old woman complained of edema and mild pain in the right LL on the fourth postoperative day after dermolipectomy. She was wearing anti-thrombosis compression stockings but had not been given pharmaceutical prophylaxis. She had a history of a left CIV stent (Figure 1) implanted by a different team 5 years previously after MTS had been diagnosed as an incidental finding in routine tests, and she had undergone a gastric bypass 3 years previously.

**Figure 2:**
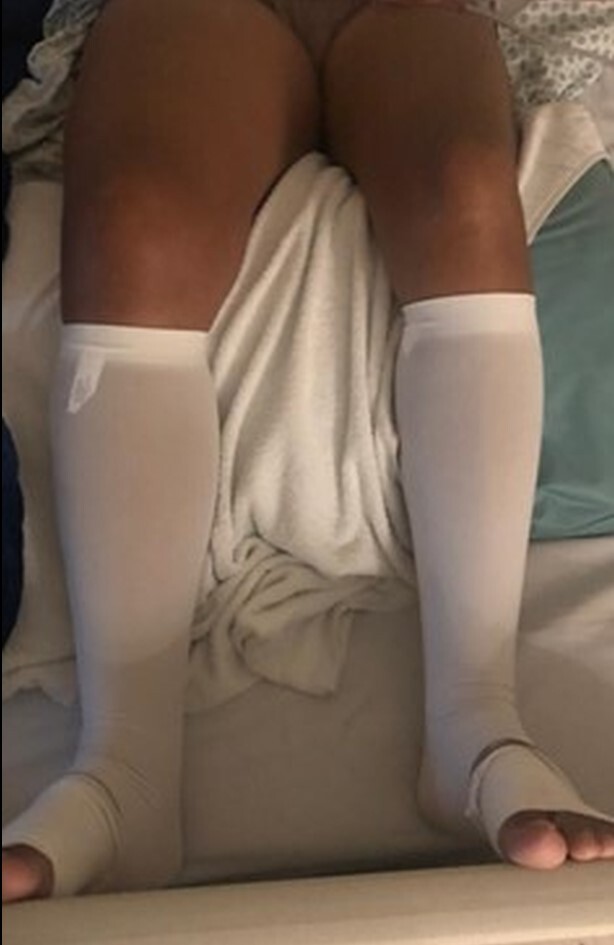
Accentuated edema of the right lower limb with no signs of venous hypertension.

**Figure 3:**
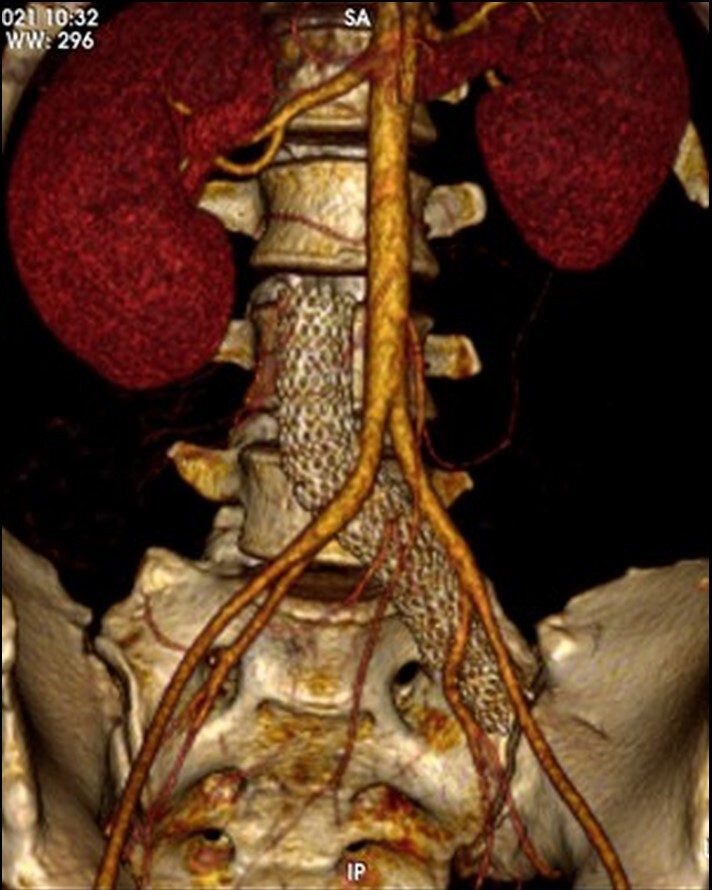
3D computed tomography angiography showing the venous stent crossing the iliocaval confluence.

On physical examination, her limbs were warm and well perfused and she had considerable edema of the right LL, but no signs of venous hypertension (Figure 2). Venous Doppler ultrasound and computed tomography angiography were ordered, showing occlusion of the right common iliac, external iliac, common femoral, and superficial femoral veins and revealing that the previous stent had been implanted beyond the recommended 2 to 3 cm, invading the IVC (Figure 3).

## PART II - TREATMENT

**Figure 4:**
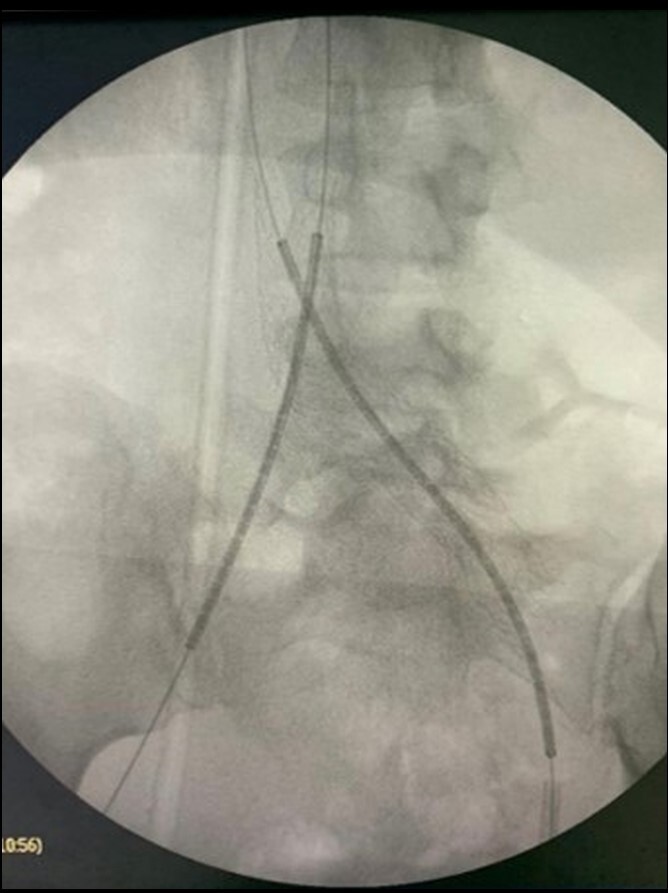
Placement of the new stents through the mesh of the previous stent.

**Figure 5:**
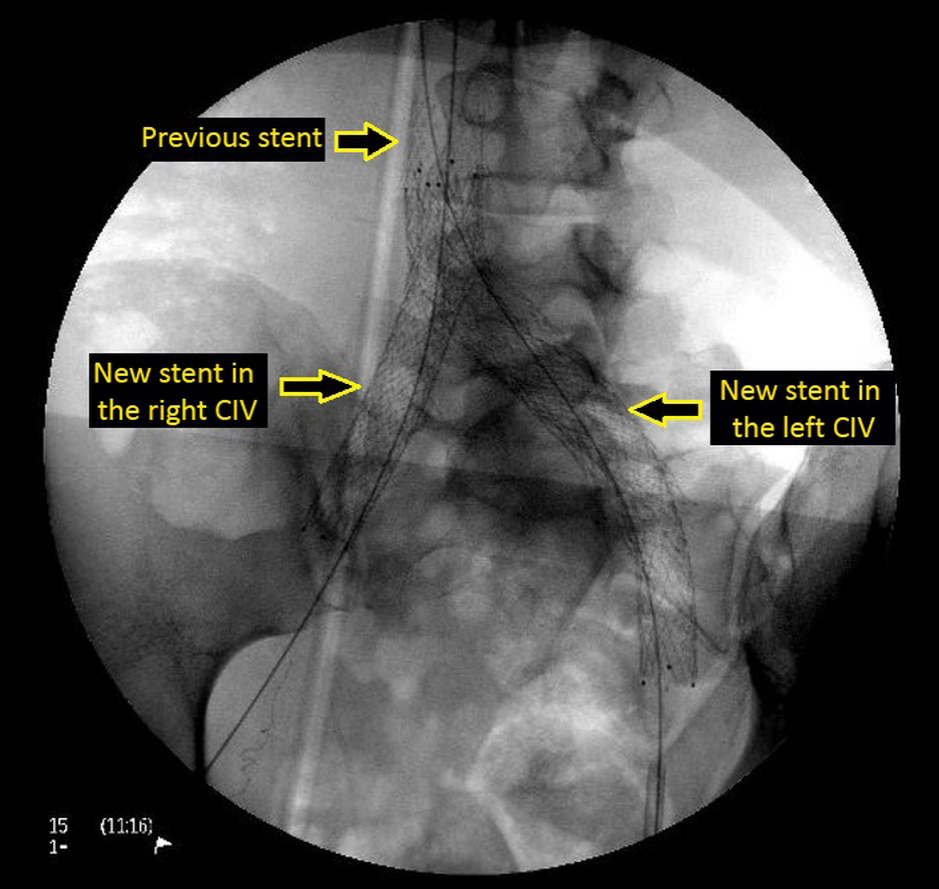
Stents deployed in the right and left common iliac veins. CIV: common iliac vein.

**Figure 6:**
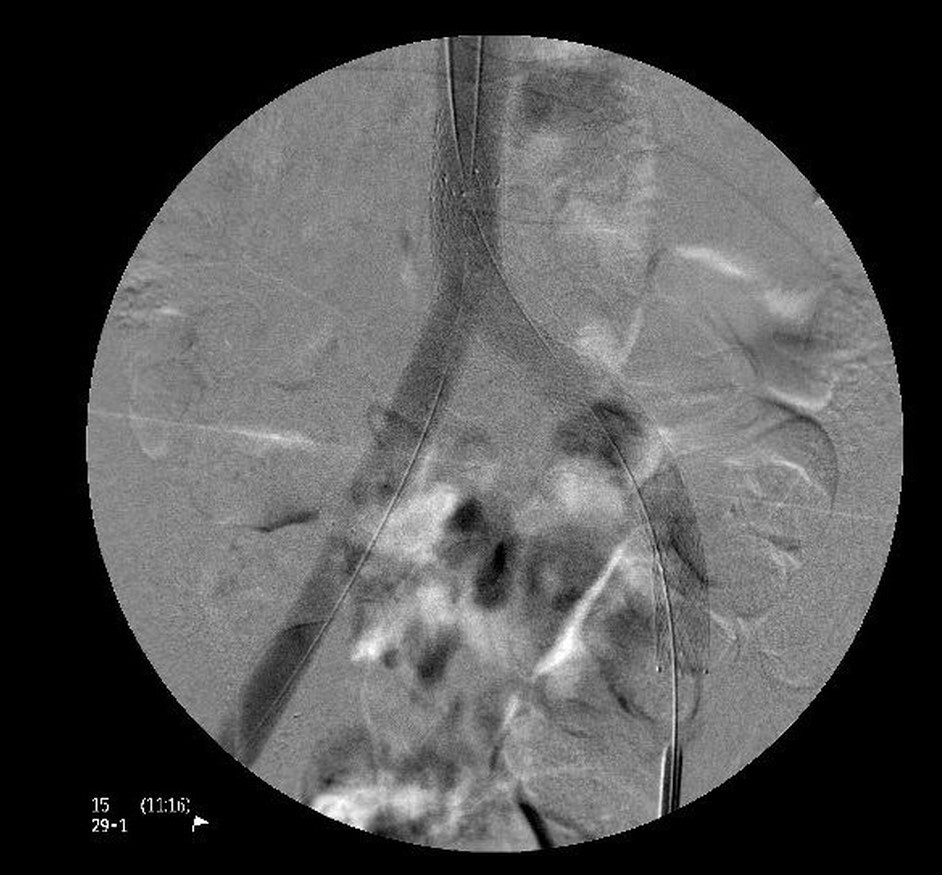
Phlebography demonstrating patency of stents.

**Figure 7:**
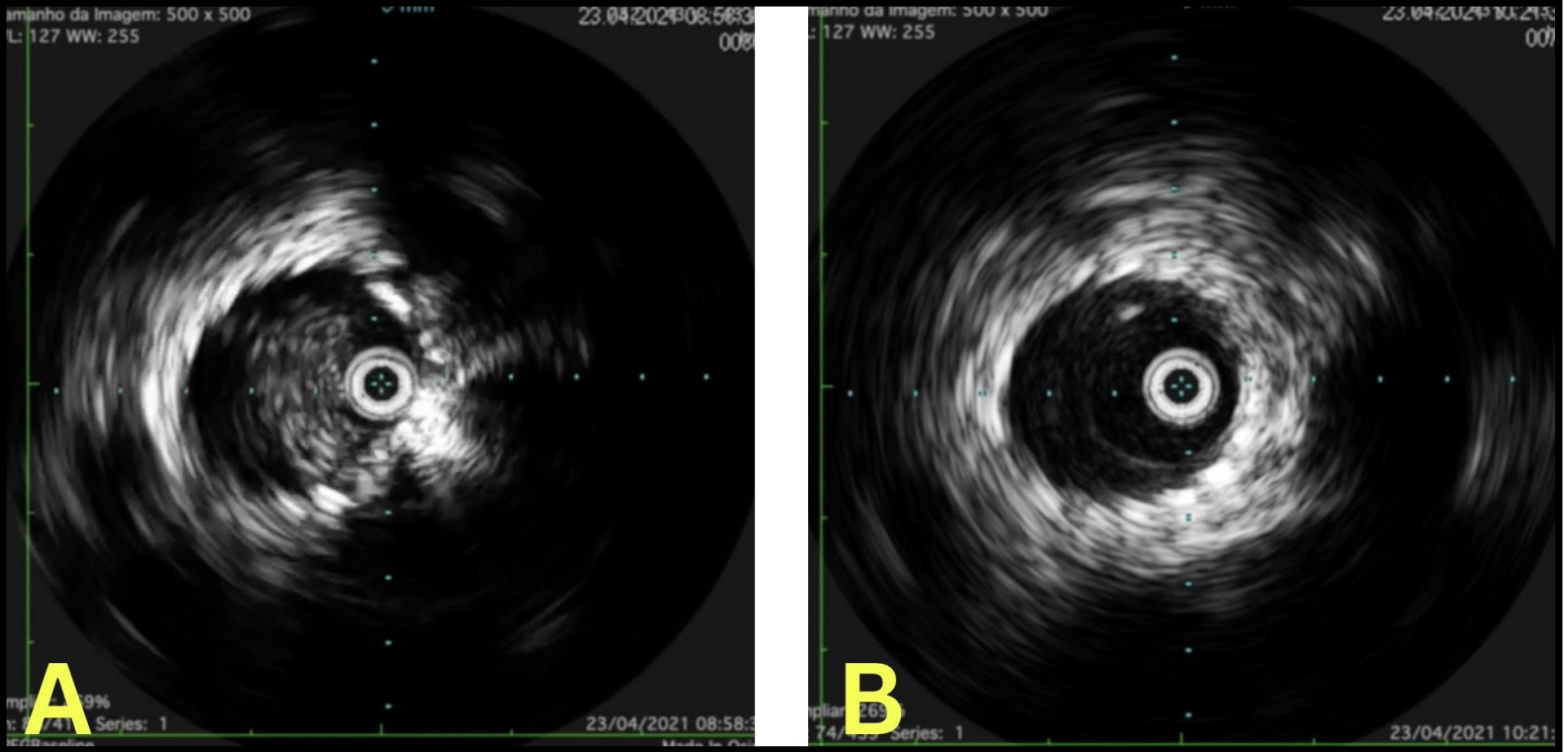
A: Intravascular ultrasound of the left common iliac vein demonstrating a previous pre-thrombectomy stent. B: Intravascular ultrasound of the left common iliac vein demonstrating free previous stent after thrombectomy.

**Figure 8:**
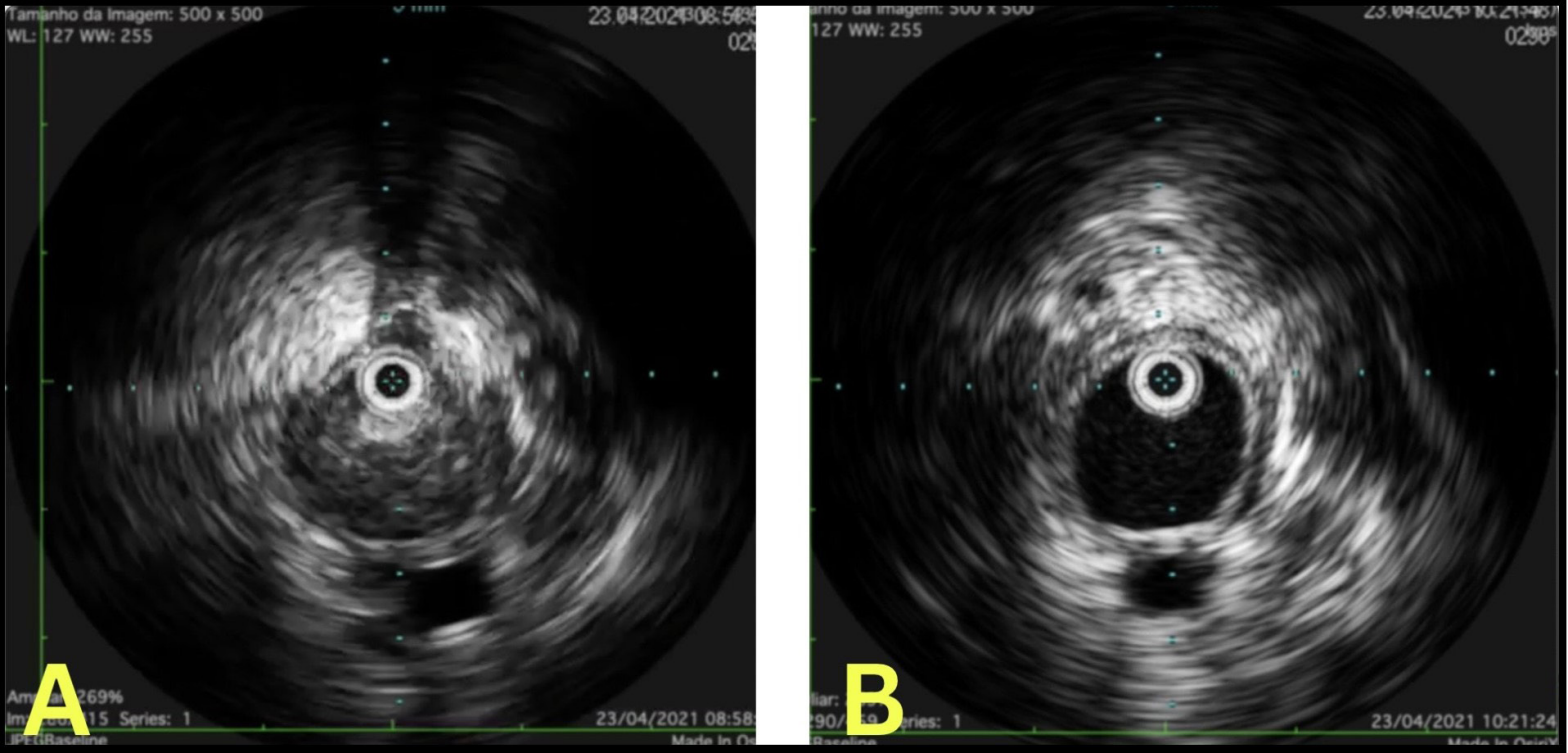
A: Intravascular ultrasound of the left external iliac vein before thrombectomy. B: Intravascular ultrasound of the free left external iliac vein after thrombectomy.

Pharmacomechanical thrombectomy was performed using an AngioJet 8 *Fr.* (Boston Scientific) with the pulse-spray technique, using 20 mg of alteplase, followed by 350 aspiration cycles. A 3 mm x 40 mm angioplasty balloon was then deployed through the mesh of the previous stent, in order to enable the intravascular ultrasound (IVUS) to be used to assess the caliber of the veins and the success of the thrombectomy procedure (Figures 4-5). Semi-complacent angioplasty balloons were then used in both common iliac veins, employing a size 14 mm x 40 mm balloon in the right iliac vein and a 16 mm x 40 mm balloon in the left, followed by placement of new stents into the right and left CIV using the double barrel technique (Figure 6) to reconstruct the iliocaval confluence (IC). VENOVO Venous Stent System™ (BD Medical, Arizona, USA) stents were used in sizes 14 mm x 100 mm, in the right CIV, and 14 mm x 140 mm in the left CIV. The stent on the right was deployed through the mesh of the stent on the left, which had been positioned first. Angioplasty to fit the stents in place was performed using a 14 mm x 40 mm balloon on the right and a 16 mm x 40 mm balloon on the left (Figure 7). Phlebography demonstrated that the stents were patent (Figure 8).

The patient was discharged from hospital two days after surgery, with significant improvements in her symptoms, including the edema, on full dose enoxaparin and dual platelet antiaggregation for the first 30 days. After this period, she continued taking enoxaparin and was put onto clopidogrel. She was followed-up for 2 months after the procedure, still on anticoagulation, and was asymptomatic with patent stents. She failed to return for follow-ups after this period.

## DISCUSSION

Endovascular implantation of a venous stent is the recommended treatment of choice for patients with symptomatic iliofemoral stenosis or occlusion and CEAP C3-C6, a moderate or severe Villalta score, and at least 50% narrowing of the lumen on venography, computed tomography, magnetic resonance, and/or IVUS.^3^

This is a safe and effective treatment for restoring venous flow, preventing DVT recurrence, reducing the risk of postthrombotic syndrome, and ensuring venous patency over the long term.^6,7^

However, in cases with proximal CIV stenosis, as in MTS, there is no consensus on the ideal placement of the stent. If the stent continues beyond the stenosis and extends into the IVC, it can compromise the contralateral flow, increasing the risk of DVT.^4^ On the other hand, if the stenosis is insufficiently covered, the stent could migrate or collapse, facilitating restenosis.^8^ There are, therefore, no precise recommendations on the length of stent that should extend into the IVC and no consensus on its clinical consequences.

Although prior studies have shown that the stent can be safely extended across the IC in the majority of patients, the reported incidence of contralateral DVT after stent placement in the CIV ranges from 1 to 15.6%.^6,8,9^

Murphy et al.^8^ highlighted two main reasons for the challenge involved in placing stents in the proximal CIV: the difficulty locating the IC with venography and the technological limitations of current stents. The first challenge can be overcome with IVUS, which offers precise visualization of the IC in 80 to 90% of cases, but the precision of stent technology remains a problem.^8^

According to Khairy et al.,^9^ factors associated with contralateral DVT after placement of a stent in the iliac vein include a history of DVT, preexisting IVC filters, failure to detect presence of compression and obstruction of the contralateral iliac vein, failure to adhere to anticoagulation, and malignancy.^9^

The rate of secondary intervention after placement of a stent in the iliac vein to treat chronic obstruction of proximal venous obstruction was 10.2% in an observational study that analyzed 490 patients and in three patients there were intraoperative findings of malpositioning beyond the recommended location and stent angulation.^10^

The case we presented highlights important learning points with regard to management of thrombotic occlusion secondary to positioning of the stent beyond the recommended position in the iliac vein. Pharmacomechanical thrombectomy with an AngioJet (Boston Scientific) followed by angioplasty restored venous flow and ensured good results, since AngioJet is associated with reduced incidence of postthrombotic syndrome and DVT recurrence.^11^ Moreover, although the literature shows that preoperative computed tomography is superior for definition and precise classification of obstructions,^12^ IVUS can also be used to conduct assessments of the IC and collect information on the degree and length of obstructive lesions, which is of particular importance for choosing the length, size, and positioning of the stents to be inserted during angioplasty, avoiding additional complications.^13^

Performing angioplasty with the double barrel technique allows reconstruction of the iliocaval confluence that had been compromised by the stent extending into the vena cava beyond the correct position. VENOVO Venous Stent System™ (BD Medical, Arizona, USA) stents are considered stable during release and have greater radial strength than other options. Their open cell design affords sufficient flexibility to accommodate movement of the hip joints without tapering, yielding excellent short term patency results and low reintervention rates.^14^

With regard to anticoagulation, we initially opted for enoxaparin because of the patient’s gastric bypass history and its detrimental effect on gastrointestinal absorption of oral anticoagulants. There is not yet consensus on the ideal duration of anticoagulation and use of platelet antiaggregants, but a minimum of 3 months of anticoagulation is recommended.^15^

## CONCLUSIONS

Although contralateral DVT after venous stenting for MTS is a rare complication, there are descriptions in the literature, primarily when associated with placement of the stent more than 2 to 3 cm beyond the recommended position. Use of IVUS is indispensable for successful deployment and correct positioning of the device. The technique described here can be used to treat this complication.
