# Supplementary material for: Deep venous thrombosis of the contralateral iliac vein after stenting of the iliocaval confluence: a therapeutic challenge
Source: J Vasc Bras. 2023 Jun 30;22:e20220162. doi: 10.1590/1677-5449.202201621 (PMC10421570; doi:10.1590/1677-5449.202201621)
Supplement: Translation

# Translation: Trombose venosa profunda de veia ilíaca contralateral após stent venoso posicionado na confluência ilíaco-cava: um desafio terapêutico

**DOI:** 10.1590/1677-5449.202201622

**Authors:** Annata Teixeira Della Costa, Igor Rafael Sincos, Lorrane Vieira Siqueira Riscado

**Affiliations:** 1 Faculdade de Medicina de São José do Rio Preto, São José do Rio Preto, SP, Brasil.; 2 Universidade de São Paulo - USP, São Paulo, SP, Brasil.; 3 Universidade Federal de Juiz de Fora - UFJF, Juiz de Fora, MG, Brasil.

**Keywords:** trombose venosa profunda, angioplastia, síndrome de May-Thurner, veia ilíaca

## Abstract

O tratamento de escolha para pacientes com síndrome de compressão venosa sintomática é o implante de stent venoso. Entretanto, esse tratamento possui complicações bem documentadas e, embora rara, trombose venosa profunda contralateral é uma dessas complicações. Nosso objetivo é apresentar um caso de trombose venosa profunda da veia ilíaca contralateral como resultado do posicionamento do stent além do preconizado e o desafio terapêutico que é a recanalização da veia com reconstrução da confluência ilíaco-cava.

## INTRODUÇÃO

A síndrome de May-Thurner (SMT) é uma condição vascular relativamente rara em que a veia ilíaca comum (VIC) esquerda é comprimida pela artéria ilíaca comum direita. Essa condição pode ser assintomática, mas, quando sintomática, apresenta sintomas de insuficiência venosa crônica (IVC) ou trombose venosa proximal, geralmente em mulheres jovens e de meia-idade. A compressão proximal da VIC é observada em combinação com IVC em até 55% dos pacientes sintomáticos^1^ .

O tratamento de escolha para pacientes com síndrome de compressão da VIC sintomática com classificação CEAP C3-C6 é o implante de stent venoso^2,3^ . No entanto, o stent deve ser implantado com precisão, pois, se for posicionado além do indicado para dentro da veia cava inferior (VCI), pode comprometer o fluxo venoso contralateral e aumentar o risco de trombose venosa profunda (TVP)^4,5^ .

O objetivo deste relato é descrever a recanalização da VIC direita por meio da realização de trombectomia fármaco-mecânica e implante de stent venoso em ilíaca comum e externa à direita em paciente com TVP extensa de membro inferior (MI) devido a stent previamente posicionado além dos 2 a 3 cm preconizados em VIC esquerda.

O protocolo foi aprovado pelo Comitê de Ética em Pesquisa da instituição (CAAE 60251422.8.0000.5226, parecer consubstanciado 5.506.965).

## PARTE I - CASO CLÍNICO

Mulher, 43 anos, com queixa de edema e dor leve em MI direito no quarto dia pós-operatório de dermolipectomia. Estava em uso de meias compressivas antitrombo e não realizou profilaxia medicamentosa. Possuía histórico de implante de stent em VIC esquerda há 5 anos (Figura 1), realizado por outra equipe, após achado de SMT em exames de imagem de rotina, além de *bypass* gástrico realizado há 3 anos.

**Figura 1:** Angiotomografia computadorizada mostrando stent venoso em veias cava inferior e ilíaca comum esquerda.

Ao exame físico, apresentava membros aquecidos e bem perfundidos, com edema acentuado de MI direito sem sinais de hipertensão venosa (Figura 2). Foram realizados Doppler venoso e angiotomografia computadorizada, os quais evidenciaram oclusão das veias ilíaca comum, ilíaca externa e femoral comum e superficial direitas, além de posicionamento de stent prévio além dos 2 a 3 cm preconizados, invadindo a VCI (Figura 3).

**Figura 2:** Edema acentuado de membro inferior direito sem sinais de hipertensão venosa.

**Figura 3:** Angiotomografia computadorizada em 3D demostrando o stent venoso ultrapassando a confluência ilíaco-cava.

## PARTE II - TERAPÊUTICA

Realizou-se trombectomia fármaco-mecânica utilizando o dispositivo AngioJet 8 *Fr.* (Boston Scientific) com a técnica *pulse-spray*, com 20 mg de alteplase, seguido de 350 ciclos de aspiração. Na sequência, realizou-se angioplastia com balão de tamanho 3 mm x 40 mm através da malha do stent prévio, a fim de permitir a passagem do ultrassom intravascular (IVUS) para avaliar o calibre das veias e o sucesso da trombectomia (Figuras 4-5). Em seguida, realizou-se angioplastia com balão semicomplacente em ambas as veias ilíacas comuns, utilizando-se o tamanho 14 mm x 40 mm para a direita e 16 mm x 40 mm para a esquerda, seguida de implante de novos stents em VIC direita e esquerda por meio da técnica *double barrel* (Figura 6) para reconstrução da confluência ilíaco-cava (CI). Utilizou-se o stent VENOVO Venous Stent System™ (BD Medical, Arizona, USA), no tamanho 14 mm x 100 mm para a direita e 14 mm x 140 mm para a esquerda. O stent da direita foi implantado através da malha do stent previamente posicionado à esquerda. Para angioplastia de acomodação do stent, utilizou-se um balão 14 mm x 40 mm à direita e um balão 16 mm x 40 mm à esquerda (Figura 7). A flebografia demonstrou perviedade dos stents (Figura 8).

**Figura 4:** Implantação dos novos stents através da malha do stent prévio.

**Figura 5:** Stents implantados em veias ilíacas comuns direita e esquerda.

**Figura 6:** Flebografia demonstrando perviedade dos stents.

**Figura 7:** A: Ultrassom intravascular de veia ilíaca comum esquerda demonstrando stent prévio pré trombectomia. B: Ultrassom intravascular de veia ilíaca comum esquerda demonstrando stent prévio livre pós trombectomia.

**Figura 8:** A: Ultrassom intravascular de veia ilíaca externa esquerda pré trombectomia. B: Ultrassom intravascular de veia ilíaca externa esquerda livre pós trombectomia.

A paciente teve alta hospitalar no segundo dia pós-operatório, com melhora significativa dos sintomas, inclusive do edema, em uso de enoxaparina dose plena e dupla antiagregação plaquetária nos primeiros 30 dias. Após esse período, seguiu em uso de enoxaparina associada a clopidogrel. Foi acompanhada por 2 meses após o procedimento, sendo mantida em anticoagulação, assintomática e com stents pérvios. Após esse período, a paciente abandonou o acompanhamento.

## DISCUSSÃO

O implante de stent venoso endovascular foi eleito o tratamento de escolha para pacientes com estenose ou oclusão iliofemoral sintomática com CEAP C3-C6, escore de Villalta moderado ou grave e pelo menos 50% de estreitamento do lúmen na venografia, tomografia computadorizada, ressonância magnética e/ou IVUS^3^ .

É um tratamento seguro e eficaz para restaurar o fluxo venoso, prevenir a recorrência de TVP, reduzir o risco de síndrome pós-trombótica e garantir a patência venosa a longo prazo^6,7^ .

Entretanto, em casos de estenose proximal da VIC, como na SMT, não há consenso sobre a localização ideal do stent. Se o stent ultrapassar a estenose e se estender para a VCI, pode prejudicar o fluxo contralateral, aumentando o risco de TVP^4^ . Por outro lado, pode migrar ou ser comprimido se a estenose não for suficientemente coberta, facilitando a reestenose^8^ . Não há, portanto, uma recomendação precisa acerca da extensão do stent na VCI nem um consenso acerca de suas consequências clínicas.

Embora estudos anteriores tenham mostrado que a extensão do stent através da CI pode ser feita com segurança na maioria dos pacientes, a incidência relatada de TVP contralateral após implantação do stent em VIC é de 1 a 15,6%^6,8,9^ .

Murphy et al.^8^ destacam duas razões principais para o desafio de implante de stent na VIC proximal: a dificuldade de localização da CI na venografia e a limitação da tecnologia dos stents atuais. O primeiro desafio pode ser superado com o IVUS, que oferece visualização precisa da CI em 80 a 90% dos casos, mas a tecnologia de precisão dos stents ainda é uma dificuldade^8^ .

Histórico de TVP, filtros de VCI pré-existentes, não identificação da presença de compressão e obstrução na veia ilíaca contralateral, não adesão a anticoagulação e malignidade são fatores associados à TVP contralateral após implantação de stent na veia ilíaca, conforme relatado por Khairy et al.^9^ .

Em um estudo observacional recente que analisou 490 pacientes, a taxa de intervenção secundária após a colocação de stent na veia ilíaca para obstrução crônica do fluxo venoso proximal foi de 10,2%, sendo que posicionamento além do preconizado e angulação do stent foram achados intraoperatórios em três pacientes^10^ .

O caso que apresentamos destaca importantes pontos de aprendizagem no manejo da oclusão trombótica secundária a posicionamento do stent na veia ilíaca além do preconizado. A realização de trombectomia fármaco-mecânica com AngioJet (Boston Scientific) seguida de angioplastia restaurou o fluxo venoso e garantiu melhores resultados, uma vez que o AngioJet está associado à redução da incidência de síndrome pós-trombótica e recorrência de TVP^11^ . Ademais, embora a literatura aponte a tomografia computadorizada pré-operatória como superior para definição e classificação precisa da obstrução^12^ , o IVUS também é capaz de fornecer avaliação da CI e informações sobre o grau e a extensão das lesões obstrutivas, o que é de particular importância na escolha do comprimento, do tamanho e da localização dos stents inseridos durante a angioplastia, evitando complicações adicionais^13^ .

Já a realização de angioplastia utilizando a técnica *double barrel* garante a reconstrução da confluência ilíaco-cava, comprometida pela presença do stent invadindo a veia cava além do preconizado. O stent VENOVO Venous Stent System™ (BD Medical, Arizona, USA) é considerado estável durante sua liberação e tem maior força radial em relação aos demais. Seu *design* de células abertas oferece flexibilidade suficiente para acomodar o movimento na articulação do quadril sem afunilar o stent, permitindo excelentes resultados de patência a curto prazo e poucas reintervenções^14^ .

Quanto à anticoagulação, optamos inicialmente pela enoxaparina devido ao histórico de bypass gástrico e ao prejuízo da absorção gastrointestinal dos anticoagulantes orais. Não há, atualmente, consenso sobre a duração ideal da anticoagulação e do uso dos antiagregantes plaquetários, sendo recomendado, no mínimo, 3 meses de anticoagulação^15^ .

## CONCLUSÃO

Apesar de ser uma complicação rara, a TVP contralateral após implante de stent venoso devido a SMT está descrita na literatura, principalmente quando associada ao posicionamento do stent além de 2 a 3 cm do preconizado. A utilização do IVUS é imprescindível para o sucesso da técnica de liberação e bom posicionamento do dispositivo. A técnica aqui descrita pode ser utilizada para o tratamento dessa complicação.
